# Probing Water and CO_2_ Interactions at the Surface of Collapsed Titania Nanotubes Using IR Spectroscopy

**DOI:** 10.3390/molecules200915469

**Published:** 2015-08-26

**Authors:** Kaustava Bhattacharyya, Weiqiang Wu, Eric Weitz, Baiju K. Vijayan, Kimberly A. Gray

**Affiliations:** 1 Department of Chemistry, Institute for Catalysis in Energy Processes, Northwestern University , Evanston, IL 60208, USA; E-Mails: kaustava78.b@gmail.com (K.B.); wu.weiqiang@northwestern.edu (W.W.); 2 Department of Civil and Environmental Engineering, Institute for Catalysis in Energy Processes, Northwestern University, Evanston, IL 60208, USA; E-Mail: baiju@cmet.gov.in

**Keywords:** collapsed titania nanotubes, FTIR, CO_2_ adsorption, defect sites, OH stretches

## Abstract

Collapsed titania nanotubes (cTiNT) were synthesized by the calcination of titania nanotubes (TiNT) at 650 °C, which leads to a collapse of their tubular morphology, a substantial reduction in surface area, and a partial transformation of anatase to the rutile phase. There are no significant changes in the position of the XPS responses for Ti and O on oxidation or reduction of the cTiNTs, but the responses are more symmetric than those observed for TiNTs, indicating fewer surface defects and no change in the oxidation state of titanium on oxidative and/or reductive pretreatment. The interaction of H_2_O and CO_2_ with the cTiNT surface was studied. The region corresponding to OH stretching absorptions extends below 3000 cm^−1^, and thus is broader than is typically observed for absorptions of the OH stretches of water. The exchange of protons for deuterons on exposure to D_2_O leads to a depletion of this extended absorption and the appearance of new absorptions, which are compatible with deuterium exchange. We discuss the source of this extended low frequency OH stretching region and conclude that it is likely due to the hydrogen-bonded OH stretches. Interaction of the reduced cTiNTs with CO_2_ leads to a similar but smaller set of adsorbed carbonates and bicarbonates as reported for reduced TiNTs before collapse. Implications of these observations and the presence of proton sources leading to hydrogen bonding are discussed relative to potential chemical and photochemical activity of the TiNTs. These results point to the critical influence of defect structure on CO_2_ photoconversion.

## 1. Introduction

Nano-scale TiO_2_ materials have attracted scientific interest due to their unusual physico-chemical properties such as high specific surface area, ion-exchangeability, and photocatalytic activity. Interest in nano-scale titania materials has extended to one-dimensional structures including nanotubes, nanorods, and nanowires, which can now be synthesized by relatively standard methods [1,2,3,4,5,6].

In the late 1990s, Kasuga *et al.* reported the first hydrothermal synthesis of a nano-tubular structure of titania [1,2]. Researchers subsequently showed that these structures consist of hydrated dititanate (H_2_Ti_2_O_5_), trititanate (H_2_Ti_3_O_7_) [3,4,5], and H_2_Ti_4_O_9_·*x*H_2_O stoichiometries [6], as well as their Na salts. These structures can be thermally treated to improve phase crystallinity, although there is still no clear consensus on the specific conditions under which thermal treatment leads to the transformation of titania/titanates in titania nanotubes (TiNTs) to TiO_2_ (B), rutile, or brookite. Nevertheless, it is clear that the morphology of the TiNTs starts to undergo a change around 550 °C. Poudel *et al.* [7] observed a change from nanotubes to nanowire morphology at an annealing temperature of 650 °C. Tsai and Teng [8], Yoshida *et al.* [9], and Vijayan *et al.* [10] all reported a drop in surface area and pore volume with increasing temperature. The surface area of the TiNTs steadily decreases as a function of temperature for as long as the nanotube morphology is retained and then decreases sharply upon further elevation of the temperature, which results in the loss of the tubular morphology [11]. Vijayan *et al.* characterized these morphological changes to TiNTs over a range of calcination temperatures from 200 to 800 °C and, using EPR, they detailed how these changes altered charge trapping behavior and photocatalytic reactivity [10].

There is an enormous body of literature on the photocatalytic reactivity, particularly oxidation reactions, of titania and doped titania materials of various morphologies. Comparatively less is known about the details of the photocatalytic reduction of CO_2_ by titania materials, especially the molecular level mechanism(s) for this complex process. Our prior study of the adsorption of CO_2_ on the surface of TiNTs and platinized TiNTs focused on changes in adsorptive chemistry that took place when the TiNTs were subjected to reductive and/or oxidative pretreatments. We found that differences in the surface species formed, which included carbonates and bicarbonates, could be correlated with the Lewis acidity and basicity of the Ti and O on the TiNT surface [12].

Calcination temperature affects the chemistry taking place on TiNTs. Vijayan *et al.* [10] reported maximum CO_2_ conversion for TiNT calcined at 400 °C and dramatically decreasing CO_2_ photo-reduction with increasing calcination temperature, particularly above 600 °C. The collapse of the tubular nanostructure is accompanied by a diminished number of under-coordinated Ti sites and marked changes in charge trapping as characterized by EPR. Differences in the respective reactivity of TiNT and collapsed TiNT (cTiNT) with CO_2_ may be explained by differences in CO_2_ binding at the catalyst surface [10,12]. Water has been found to be a critical participant in the reduction of CO_2_. The presence of water has been shown to facilitate CO_2_ photoreductive chemistry. This is not surprising since water can provide the protons needed to go from CO_2_ to hydrocarbons and the coordination of water can potentially lower the barrier for reactions to desired hydrocarbon products such as formic acid and methanol.

The objective of the research reported herein is to spectroscopically probe the interactions of water and CO_2_ at the cTiNT surface with the ultimate goal of identifying the surface characteristics of photocatalytic materials capable of driving CO_2_ conversion. Specifically, we report the effect of higher temperature annealing on the phase, structure, and morphological distortion, which leads to changes in the chemical interactions of CO_2_ with cTiNT. We also address an issue in the literature with regard to the assignment of low frequency absorptions in the OH stretching region of the infrared. These absorptions are assigned to OH stretches that form on exposure of the nanotubes to water. We speculate that hydrogen bonding between H-containing moieties within the structure of the nanotubes may be the sources of these low frequency OH absorptions.

## 2. Results

### 2.1. X-ray Diffraction Data

**Figure 1:**
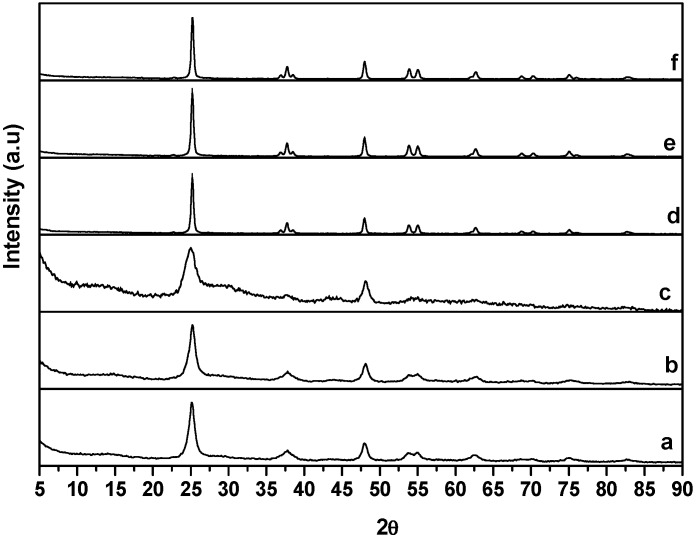
XRD profiles of (**a**) TiNT; (**b**) TiNT-O_2_; (**c**) TiNT-O_2_-H_2_; (**d**) cTiNT-; (**e**) cTiNT-O_2_; (**f**) cTiNT-O_2_-H_2_. Note that traces **a**–**c** have been included here for completeness and are reproduced from Figure 2 in reference [12]. Reprinted with permission from: Kaustava Bhattacharyya, Alon Danon, Baiju K. Vijayan, Kimberley A. Gray, Peter C. Stair, and Eric Weitz, *The Journal of Physical Chemistry C* **117** (2013), 12661–12678, Copyright 2013, American Chemical Society.

The interpretation of the X-ray diffraction data (Figure 1) for the titania nanotubes that have been heated to 350 °C has been discussed previously in detail [12]. These data confirm that the TiNTs are essentially pure anatase. Thermal annealing at 650 °C leads to formation of a rutile phase along with the characteristic anatase phase. As seen in Figure 1, after annealing at 650 °C, a series of diffraction peaks appear which are consistent with anatase TiO_2_ (JCPDS 75-1537): (101), 25.7°; (200), 47.9°; (105), 53.7°; (211), 55.0°; (213), 61.9°; (204), 62.8°; (116), 68.3°; (220), 69.7°; and (301), 75.2°. An additional diffraction peak characteristic of the rutile phase is seen at 27.3° (110) (JCPDS-77-0443, 77-0444). This new peak indicates that there is a partial transformation between anatase and rutile as a result of heating to 650 °C. Though anatase is metastable in the bulk, it has been reported that the anatase phase is more stable as nano-scale TiO_2_ than as bulk TiO_2_ [13,14,15,16,17]. A prior report for TiNT prepared by anodization of Ti foil indicated that above 550–600 °C, rutile is the dominant phase (>50%) [18,19]. In contrast, Albu *et al.* found that double-walled anodized TiNTs were extraordinarily stable, retained their structural integrity even when annealed to temperatures higher than 600 °C, and showed only traces of rutile [20]. Similarly, we determine a rutile composition of only ~5.5%. The smaller amount of rutile formed is probably due to the enhanced stability of anatase in nano-scale titania materials, but the transformation could also be kinetically limited, possibly as a result of the hydrothermal synthesis process. However, the peak at a 2θ value of 13.3° is completely absent in the XRD pattern, indicating the loss of the titania nanotubular morphology for samples calcined at 650 °C.

**Figure 2:**
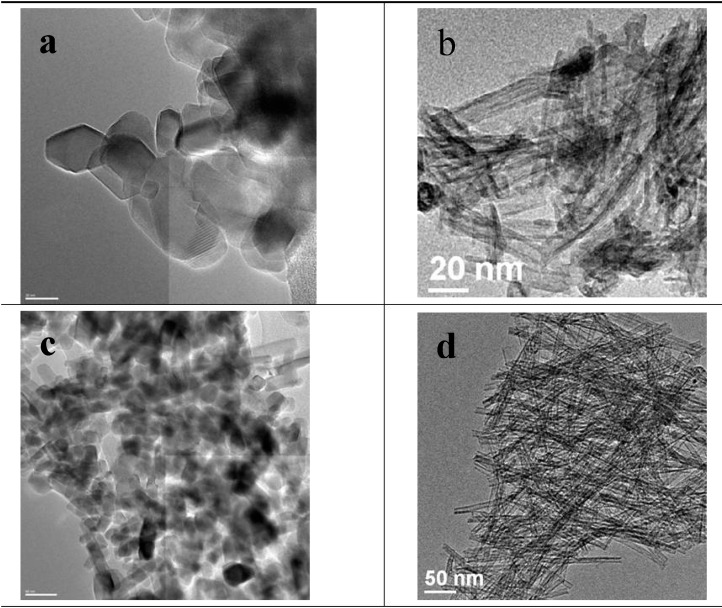
TEM images of the TiNT and the cTiNT: (**a**) cTiNT with a magnification scale of 20 nm; (**b**) TiNT with a magnification scale of 20 nm; (**c**) cTiNT with a magnification scale of 50 nm; (**d**) TiNT with a magnification scale of 50 nm. Note that panels (**b**,**d**) are included here for completeness and are from Figure 1 in Reference [12]. Reprinted with permission from: Kaustava Bhattacharyya, Alon Danon, Baiju K. Vijayan, Kimberley A. Gray, Peter C. Stair, and Eric Weitz, *The Journal of Physical Chemistry* C **117** (2013), 12661–12678, Copyright 2013, American Chemical Society.

### 2.2. Transmission Electron Microscopy

**Figure 3:**
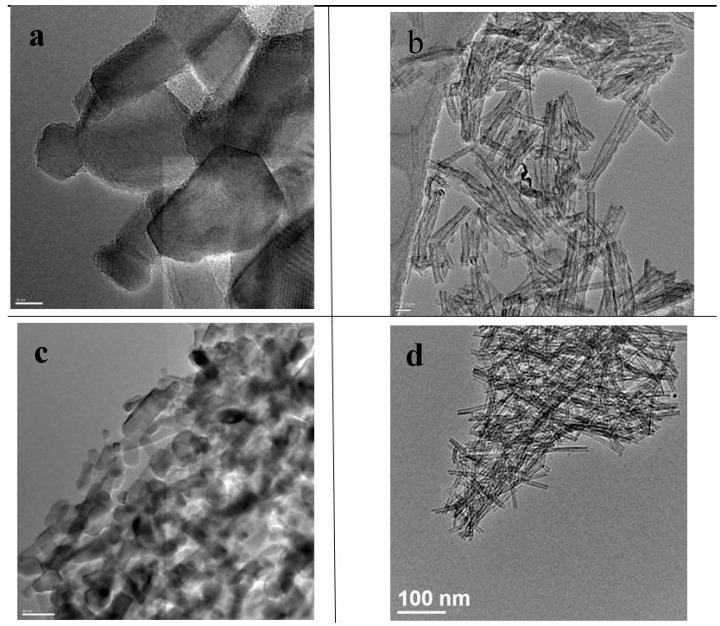
TEM images of the TiNT-O_2_ and the cTi-NT-O_2_: (**a**) cTiNT-O_2_ with a magnification scale of 20 nm; (**b**) TiNT-O_2_ with a magnification scale of 20 nm; (**c**) cTiNT-O_2_ with a magnification scale of 50 nm; (**d**) TiNT-O_2_ with a magnification scale of 100 nm.

**Figure 4:**
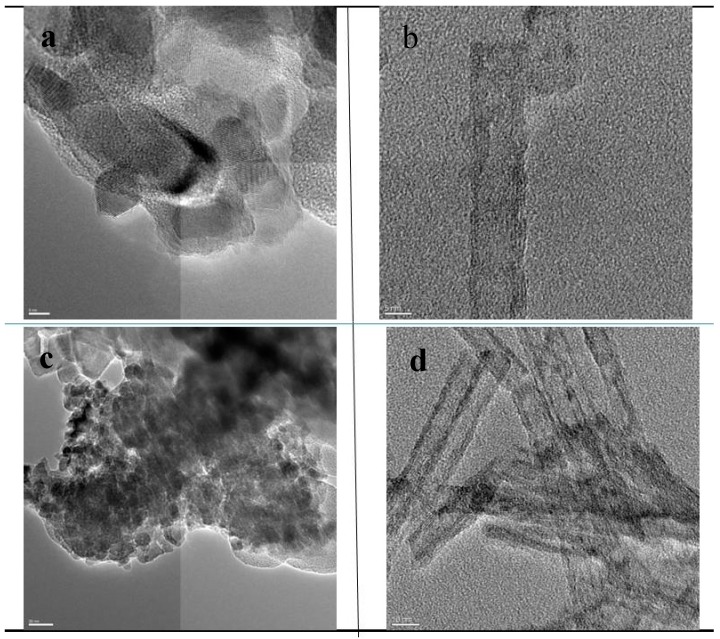
TEM images of the TiNT–O_2_-H_2_ and the cTiNT-O_2_-H_2_: (**a**) cTiNT-O_2_-H_2_ with a magnification scale of 20 nm; (**b**) TiNT with a magnification scale of 20 nm; (**c**) cTiNT-O_2_-H_2_ with a magnification scale of 50 nm; (**d**) TiNT with a magnification scale of 50 nm. Note that panel 2.3d is from Figure 1 in reference [12]. It has been included here for completeness and is reprinted with permission from: Kaustava Bhattacharyya, Alon Danon, Baiju K. Vijayan, Kimberley A. Gray, Peter C. Stair, and Eric Weitz, *The Journal of Physical Chemistry* C **117** (2013), 12661–12678, Copyright 2013, American Chemical Society.

The interpretation of the electron microscopy data for the titania nanotubes heated to 350 °C has been discussed in detail previously [12]. Briefly, the data in Figure 2, Figure 3 and Figure 4 compare HRTEM images of the TiNTs calcined at 350 °C and 650 °C. The conclusion from the HRTEM data is that the nanotubular morphology of the TiNTs is intact for the TiNTs calcined at 350 °C and after pretreatment under an oxygen atmosphere, and subsequently under H_2_. However, when these TiNT are calcined at 650 °C, the tubular morphology is lost and the tubes transform into a variety of other nanoscale shapes, although nanorods are the predominant shape.

### 2.3. BET Surface Area

Measurement involving a determination of the BET surface area and pore size distribution of TiNTs has been previously reported [10,12]. Briefly, the as-prepared TiNTs have a surface area of ~235 m^2^/g, which is almost 20 times that of the TiO_2_ anatase powder from which the tubes were prepared. Upon calcination at 350 °C the surface area decreases to ~200 m^2^/gm. When these TiNTs are subject to either oxidative and/or reductive pretreatments at 350 °C the surface area decreases further, into the ~180–195 m^2^/gm range, which is still comparable to that before pretreatment. Upon calcinations of the TiNTs at 650 °C the surface area decreases substantially to ~90 m^2^/g. The surface areas do not change significantly for the cTiNT samples as a result of oxidization and subsequent reduction. Thus, as the temperature increases toward 650 °C, the collapse of the nanotubes leads to formation of coarser grain particles and as the crystallite size grows, so does the inter-particle pore size.

### 2.4. XPS

**Figure 5:**
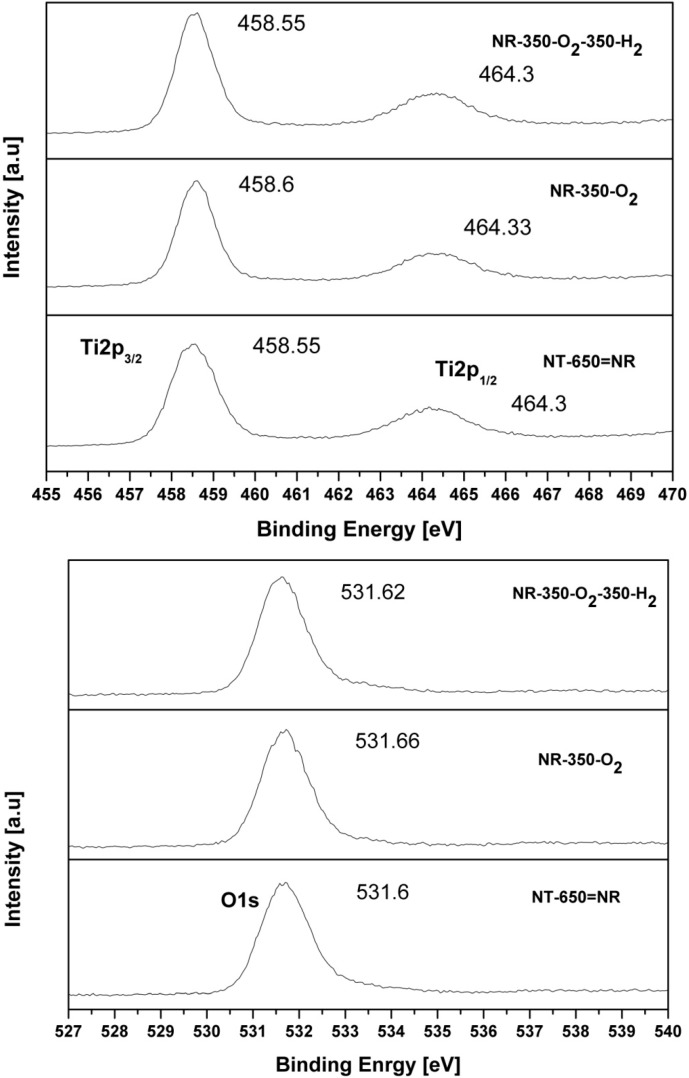
The XPS spectra for (**top panel**) Ti 2p and (**bottom panel**) O 1s for the cTiNTs which have been subjected to different pre-treatments: (**bottom trace**) cTiNT; (**middle trace**) cTiNT-O_2_; (**top trace**) cTiNT-O_2_-H_2_.

The top panel in Figure 5 shows the XPS Ti 2p spectra of the cTiNT subjected to oxidative and reductive pretreatments. All of the cTiNT samples have Ti 2p_3/2_ and Ti 2p_1/2_ peaks at 458.6 and 464.3 eV, respectively, in their Ti XPS spectra. These peak positions are typical of anatase TiO_2_ nano-powder samples [21,22]. There is no observed change in these peaks as a function of oxidation or reduction. The lack of change in these peaks on oxidation and/or reduction is in marked contrast to what is observed for TiNT samples, where upon reduction there is formation of the Ti^3+^ and splitting of the Ti^4+^ peak [12].

The O 1s XPS spectra shown in the bottom panel in Figure 5 for the samples calcined at 650 °C (cTiNT) exhibits analogous behavior. Each sample shows an O 1s XPS peak at 531.66 eV, which is typical of bulk anatase TiO_2_ samples. These O 1s peaks are symmetric and possess a smaller FWHM than the TiNT samples calcined at 350 °C, which is indicative of fewer O defects and fewer OH groups. Thus, we can infer based on the XPS data [21,23] that the electronic environment of surface Ti and O atoms in the nanotubular morphology is more sensitive to oxidation and/or reduction than their counterparts in the nanotubes that have collapsed as a result of annealing. Overall, there is no observable shift in either the Ti or the O XPS peaks upon oxidation of the cTiNT, and upon reduction there is neither formation of Ti^3+^ for this sample nor is there any splitting of the Ti^4+^ peak, as was observed for the corresponding TiNTs that were annealed at 350 °C [12].

### 2.5. FT-IR Spectroscopy

**Figure 6:**
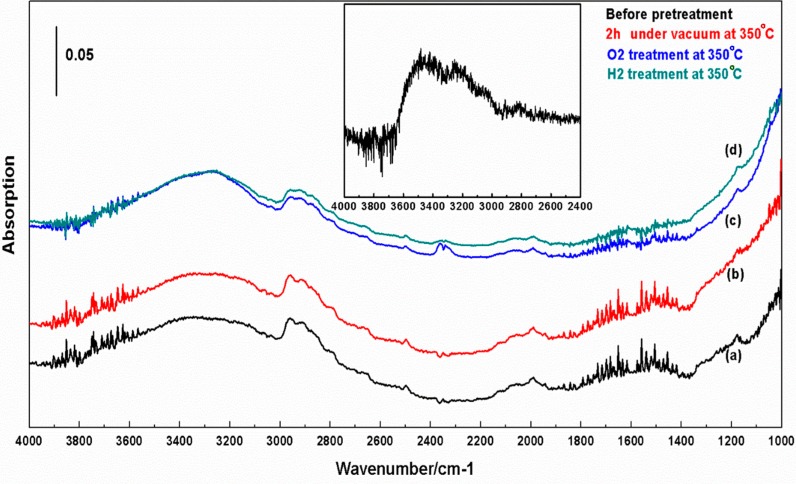
*In situ* FT-IR spectra of the c-TiNT. The collapsed NTs were prepared by calcination at 650 °C in a furnace and were allowed to cool to room temperature and introduced into the IR cell and an IR spectrum was recorded (**a**). Spectrum **b** was recorded after these cTiNTs were exposed to vacuum for 2 h at 350 °C. Spectrum **c** was recorded after the same cTiNTs were heated in O_2_ for 3 h at 350 °C to produce cTiNT-O_2_. Spectrum **d** was recorded after the oxygen was evacuated from the cell subsequent to obtaining spectrum **c** and the oxidized cTiNTs were heated in H_2_ for 3 h at 350 °C to produce cTiNT-O_2_-H_2_. The inset shows a cTiNT-O_2_ sample that has been exposed to 10 Torr of water vapor for 15 min at room temperature. The background for all spectra was the cell with the tungsten wire grid in place but no sample on the grid.

Figure 6 shows a spectrum of the cTiNTs after they have been collapsed by calcining to 650 °C and then cooled to room temperature. There is an absorption that stretches from ~3700 cm^−1^ down to ~2700 cm^−1^. The absorption appears to consist of at least two overlapped peaks as a result of the increase in intensity near 3000 cm^−1^. There are no observable absorptions due to Ti-OH stretches, which are expected near 3700 cm^−1^ [24]. The insert shows the cTiNT-O_2_ after exposure to water vapor at room temperature and subsequent to evacuation of the water vapor.

**Figure 7:**
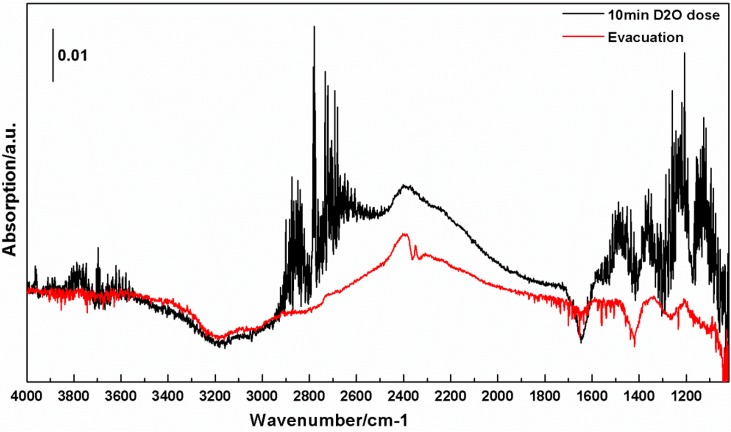
The upper spectrum is the *in situ* FT-IR spectra of the cTiNT-O_2_-H_2_ after exposure to D_2_O vapor for 10 min at room temperature. The lower spectrum is of the same sample after exposure to vacuum at room temperature. The background for these spectra is the c-TiNT-O_2_-H_2_s.

To probe the source of these absorptions we exposed the sample to D_2_O. Figure 7 shows that exposure to 10 Torr of gas-phase D_2_O produces a broad absorption between ~2700 and ~1950 cm^−1^. A “negative absorption” is also seen between ~3500 and ~2700 cm^−1^. We note that the spectra shown are referenced to background spectra of the cTiNTs. Thus, depletion of a species that is present and absorbs in the background spectrum will appear as a “negative absorption” in the sample spectrum. The upper trace also has absorptions due to OD stretching modes of D_2_O in the ~2900 to ~2500 cm^−1^ range, which show the rotational structure characteristic of gas-phase absorptions, as do the absorptions below 1600 cm^−1^, which are due to the bending mode of D_2_O. The lower trace demonstrates that evacuation removes the gas-phase D_2_O. Evacuation also leads to a decrease in intensity of the 1950 to 2700 cm^−1^ absorption, with a larger decrease in intensity at higher frequencies, where weakly bound D_2_O would be expected to absorb. There is also a sharp peak at 2350 cm^−1^ that is superimposed on the broad absorption. This is where gas-phase CO_2_ absorbs [25], and this absorption is likely due to a small change in the CO_2_ content of the purged housing that takes place over the timescale of the experiment.

**Figure 8:**
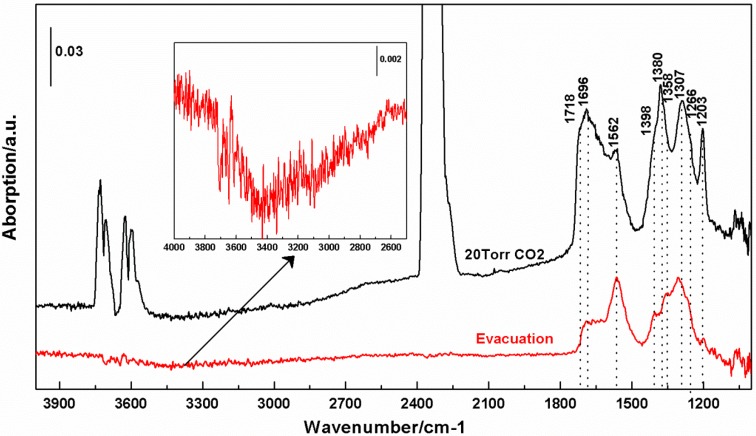
The upper trace is an *in situ* FT-IR spectrum of the cTiNT-O_2_-H_2_ exposed to 20 Torr of CO_2_ for 30 min at room temperature. The lower trace is the same sample after exposure to vacuum at room temperature. The background for these spectra is the c-TiNT-O_2_-H_2_. The insert is a blow-up of the 4000 to ~2400 cm^−1^ region.

Figure 8 shows the spectrum obtained on exposure of the reduced cTiNTs to 20 Torr of CO_2_. The very intense absorption centered at 2350 cm^−1^ is due to the asymmetric stretch of CO_2_ and the absorptions near ~3610 and ~3715 cm^−1^ are due to combination bands of CO_2_ [25]. Carbonates and bicarbonates have characteristic absorptions below ~1700 cm^−1^ [26]. As shown in the insert, exposure to CO_2_ also leads to a small “negative” absorption in the OH stretching region. The nature of these absorptions along with the absorption at ~1718 cm^−1^ is discussed in more detail below.

## 3. Discussion

### 3.1. Structure of the NTs

It is clear from the XRD results that as the TiNTs are annealed to 650 °C, the anatase TiNTs are partially transformed to the rutile phase. The TEM results show that the tubular morphology is completely lost upon calcining at 650 °C. In contrast to XPS data showing significant changes in the electronic environment around the Ti and O sites on oxidation and/or reduction of the TiNTs heated to 350 °C [12], the XPS spectra shown in Figure 5 reveal that there are no significant changes in the electronic environment around the Ti and O surface sites on oxidation and/or reduction of the cTiNT. All of the XPS spectra for the cTiNTs exhibit Ti 2p_3/2_ and Ti 2p_1/2_ peaks at 458.6 and 464.3 eV, respectively. They also have very similar O 1s XPS spectra, which are not affected by further oxidation or reduction. Each spectrum shows an O 1s XPS peak at 531.6 eV that is typical of bulk anatase TiO_2_ samples. These O1s peaks [27] are symmetric and possess a smaller FWHM than seen for the TiNT samples that were calcined at 350 °C [12]. The narrowing and the symmetry of this peak are likely the result of either fewer surface –OH groups, O defect sites, non-stoichiometric O sites, or some combination of the three [23,27]. One of the more interesting observations with the TiNT samples is that reduction subsequent to oxidation leads to the appearance of a XPS peak that is characteristic of Ti^3+^ [12]. No Ti^3+^ signals are seen for any treatment of the cTiNT samples that we have studied.

### 3.2. Interactions of H_2_O and D_2_O with c-TiNT

Titania nanotubes are formed from the dehydration of titanates, such as H_2_Ti_2_O_5_, that is driven by calcination at elevated temperatures. Figure 6 shows the IR spectrum of cTiNT (calcined at 650 °C in an oven, allowed to cool to room temperature, and then transferred to our IR cell). Traces are shown for the cTiNTs after being transferred into the cell, after 2 h under vacuum at room temperature, after oxidation by heating in oxygen at 350 °C for 3 h, and after reduction in hydrogen at 350 °C for 3 h (subsequent to oxygen pretreatment and evacuation of oxygen). The high energy portion of the four spectra are dominated by a broad absorption starting near ~2700 cm^−1^ with a feature with maximum amplitude near 2950 cm^−1^ and another broad feature that peaks in the 3300 to 3400 cm^−1^ region.

There have been many studies of water adsorption on metal oxides and on titania in particular. Suda and Morimoto report three features above 3000 cm^−1^ on rutile titania powder [28]. One is a sharp absorption near 3660 cm^−1^, which is split into a doublet at elevated temperature. This feature is assigned to surface OH groups, and the two components of the peak are attributed to monodentate and bidentate OH binding. The remainder of the absorption above 3000 cm^−1^ is assigned to surface-bound water. They assign the feature at 3520 cm^−1^ to hydrogen-bonded OH groups and the broad feature at ~3400 cm^−1^ to molecular water. The interaction of water with a thin titania film was studied by Nakamura *et al.*, and a broad absorption due to physisorbed water is reported centered at 3500 cm^−1^ [29]. This water can be removed by evacuation at room temperature. More strongly absorbed water is reported to be centered at 3270 cm^−1^. This assignment parallels those for other metal oxides where physisorbed water is reported at higher frequencies than more strongly adsorbed water and both absorb above 3000 cm^−1^ [24]. Ti-OH absorptions, if present, are typically reported around 3700 cm^−1^ [24]. Interestingly, a spectrum reported by Tsuchiya *et al.* [30] for anodic titania NTs shows a broad absorption centered at 3450 cm^−1^ that extends below 3000 cm^−1^. Chen *et al.* [5] also present a spectrum of titania NTs that shows a broad absorption from just above 2500 cm^−1^ to ~ 3400 cm^−1^.

The spectra reported by Tsuchiya *et al.* [30] and Chen *et al.* [5] are qualitatively similar in extent and shape to the water absorption shown in Figure 6. There is no absorption characteristic of OH groups in Figure 6 (or in the two references cited above). This is consistent with the removal of OH groups from the surface when the TiNTs are heated to 650 °C. Presumably this can occur as a result of a reaction to form water. This water then either desorbs into the gas phase or adsorbs on the cTiNTs. Water can also adsorb on the cTiNTs as they cool in air. Gas-phase water has its asymmetric stretching vibration centered at 3756 cm^−1^, its symmetric vibration centered at 3651 cm^−1^, and its bending mode centered at 1595 cm^−1^ [25]. The manifold of small peaks between 3900 and 3500 cm^−1^ and between 1400 and 1900 cm^−1^ in Figure 6 are due to absorptions of gas-phase water. These absorptions are likely due to small changes in water content in the purged spectrometer housing taking place over the timescale of hours. Inspection of Figure 6 clearly demonstrates that adsorbed water absorbs at a lower frequency than gas-phase water. The observation that there is little change in the shape of the higher energy portion of the water absorption upon evacuation at an elevated temperature (350 °C) is consistent with the dominant water species that is present being strongly adsorbed water, which absorbs at a lower frequency than weakly bound water [24]. We now turn to the lower frequency portion of the absorption in this region.

Absorptions at lower frequencies than seen for adsorbed water on anatase powder or thin films were noted in the literature [5,30] and in a prior study of TiNTs in our group [31]. In that study, TiNTs decorated with Pt nanoparticles were exposed to supra-bandgap light [31]. Pt nanoparticles can act as a “sink” for photo-generated electrons, extending the lifetime of photo-generated holes. The longer hole lifetime leads to greater oxidation of water. On illumination of the platinized TiNTs, the intensity in the region near 3000 cm^−1^ decreases below the baseline. Again, we note that a “negative absorption” is characteristic of a depletion of a species that is present in the background spectrum. This depletion on illumination of the platinized TiNTs is preferentially in the low frequency region of the water absorption and suggests that this lower frequency region is dominated by a different type of water than water absorbing at higher frequencies. Since the holes are primarily expected to be generated within the Pt-TiNTs, the water that they react with leading to preferential depletion of the low frequency region of the absorption near 3000 cm^−1^ was referred to as “structural water”. This term is meant to indicate water within the structure of the TiNT, where it may be more proximate to the location of hole generation than surface water. The depletion of structural water is accompanied by an increase in the absorption at higher frequencies as more surface-bound water is produced as a result of H and OH thus generated with other OH groups [31]. Thus, our findings are consistent with previous work and suggest that there are two different OH stretching regions.

We note that the shape of the spectrum in the water region in Figure 6 is also consistent with two absorption regions: one that dominates the high frequency region and the other that dominates the low frequency region. The lower frequency band has a maximum near 2900 cm^−1^ and a relatively sharp drop-off near 3000 cm^−1^. The partitioning of amplitude between these two bands is affected by the treatment and history of the cTiNTs. Though we have not studied this effect in detail, as discussed below, there are reports in the literature that protons are incorporated in the TiNT structure. This is expected based on the exchange of Na^+^ with protons during the TiNT synthesis. This could result in a change in the effective pH of the interior of the TiNTs and lead to a change in the hydrophilicity of the interior, making it a more favorable environment for “structural water”. On collapse, some of this water and/or protons could be trapped in the interior of the cTiNTs.

The data in Figure 7 show the spectrum of the collapsed oxidized cTiNTs after exposure to gas-phase D_2_O. New absorptions as a result of interaction with D_2_O are seen in the 1950–2700 cm^−1^ region and loss of absorption is seen in the 2700–3500 cm^−1^ region. It is difficult to provide an exact delineation of the extent of either region because of uncertainties in the baseline. However, a “negative absorption” is seen between ~3550 and ~2700 cm^−1^. These spectra are referenced against a background spectrum of the cTiNT-O_2_-H_2_s. A negative absorption indicates that a species that was present in the background is no longer present in the displayed spectrum. New absorptions are seen between ~2700 and ~1950 cm^−1^. A new absorption with rotational structure is seen between ~2500 and ~2900 cm^−1^. The asymmetric stretching mode of gas-phase D_2_O is centered at 2789 cm^−1^ and the symmetric stretch is centered at 2666 cm^−1^. A Q branch is apparent at 2789 cm^−1^. Thus, the new absorption with rotational structure centered at ~2789 cm^−1^ is due to stretching modes of gas-phase D_2_O. The ratio of the frequencies of the vibrational modes of gas-phase D_2_O and their protonated counterparts is calculated from their spectra to be between ~0.73 and 0.74 [25]. A value of ~0.73 would be predicted from the ideal harmonic oscillator model for an OH stretch. There is a wider spread in these ratios for water absorptions in solid hydrates [32]. The ratio of the frequencies of the high and low energy edges of the loss in absorbance (negative absorption) and the new absorption is ~0.76 and ~0.72. Given the uncertainties as to where the two regions begin and end, and potential perturbations due to the difference in the environment in these experiments and the gas phase, we conclude that these frequency ratios are consistent with deuterated water exchanging with protonated water. The fact that the entire absorption band above 2700 cm^−1^ is depleted on interaction with D_2_O is strong evidence that this absorption is due to moieties whose protons are readily exchanged by exposure to D_2_O and, thus, this observation is consistent with absorptions being due to OH stretching modes.

The question remains, though, why do we see absorptions from OH stretching modes at a significantly lower frequency than the OH stretches in water adsorbed on the surface of titania powders? We do not have a definitive answer, but there is considerable evidence in the literature that interactions in the solid state can lead to shifts in OH stretching frequencies. The OH and OD stretching frequencies for solid hydrates can be significantly red-shifted [32]. Hydrogen bonding can lead to the appearance of an absorption that is at a lower frequency than a typical OH absorption. The length of the hydrogen bond has been correlated with its absorption frequency, and has been reported to be as low as 2000 cm^−1^ [33]. The exact structure of TiNTs is still unresolved, and may be influenced by the synthesis procedure and subsequent treatments. The cTiNTs have been subjected to much less detailed study. However, evidence has been presented in the literature to show that TiNTs can contain water and protons [34,35]. This opens up the possibility for hydrogen bonding within the structure of TiNTs. The structure of the cTiNTs is different than those of the multi-walled TiNTs; however, the similarity between what we observe and what is observed in prior studies [5,30] suggests similar factors leading to a lower frequency band in the region of the spectrum in which OH absorptions appear. This hydrogen bonding would involve water and/or protons. It is possible that the cTiNTs trap water and/or protons within their structure on collapse of the TiNTs. It is also likely that the cTiNTs adsorb atmospheric water as they cool after being collapsed in an oven.

### 3.3. Interactions of CO_2_ with cTiNT

Figure 8 shows the spectrum taken after exposure of the cTiNT-O_2_-H_2_ to CO_2_. The new absorptions in the 1700 to 1200 cm^−1^ region are characteristic of carbonates and bicarbonates [12,26,31]. Though the exact nature of the observed carbonate or bicarbonate species is not of major significance in the current study, for completeness we provide the assignments we have deduced for the observed absorptions with the caveat that some of these assignments are still a subject of discussion [12,26,31].

- Bidentate carbonate (in cm^−1^): 1690, 1562, 1380, 1358, 1307
- Monodentate carbonate (in cm^−1^): 1266
- Bicarbonate (in cm^−1^): 1404, 1398, 1203
- The 1290 cm^−1^ absorption is likely a convolution of peaks including a bidentate carbonate reported at 1278 cm^−1^.

We note that many of the absorption features are lost on evacuation, which is consistent with weakly bound species. The peak frequencies of the remaining absorptions may then shift slightly due to overlap with other absorptions that disappeared on evacuation. Evacuation results in bidentate carbonate absorptions at 1563, 1358, and 1307 cm^−1^, a bicarbonate at 1404 cm^−1^, and a monodentate carbonate at 1266 cm^−1^. The absorption at 1718 cm^−1^ is too high in energy to be due to a carbonate or bicarbonate. It is in a region in which carbonyl stretching absorptions can appear [36]. The carbonyl stretch of gas-phase formic acid absorbs at 1740 cm^−1^ [25] and an absorption due to formic acid has been assigned previously for an uncollapsed platinized TiNT at ~1725 cm^−1^ [31]. Thus, we assign the 1718 cm^−1^ absorption to the carbonyl stretch of formic acid. The observation of formic acid is interesting since there are reports of dissociative adsorption of formic acid to formate for anatase titania, particularly for the (101) plane [37]. A search of the literature does not provide a definitive explanation for our observation of formic acid. However, we note that formate and formic acid are expected to be in equilibrium. The observation of formate on exposure to formic acid suggests that in those systems, the equilibrium is heavily shifted toward formate. Since titania provides an acidic surface [12] and our infrared studies provide evidence for the presence of protons and/or water, we hypothesize that in our system, the presence of proton sources is sufficient to shift the equilibrium for the nanotubes towards formic acid. Other factors could also contribute to our observation of formic acid. One suggested pathway for formic acid formation involving isolated hydroxyls [38] would not be expected to be significant in our system due to the absence of isolated OH moieties. It is also possible that the efficient sites for dissociation of formic acid in the presence of water, such as the (101) plane [37,39], have been annealed as a result of high temperature calcination of the samples under study.

Clearly there is less adsorption due to carbonates and bicarbonates than seen with the uncollapsed TiNTs. A decrease in absorbance would be expected due to a decrease in surface area of the cTiNTs *versus* the uncollapsed TiNTs. There is also a difference in the pattern of the absorptions seen relative to that for the reduced TiNTs. However, what is perhaps most surprising is the degree of similarity in the absorptions between the similarly treated collapsed and uncollapsed reduced TiNTs, though there are more species absorbing for the reduced uncollapsed TiNTs. The observation of formic acid suggests that these cTiNT-O_2_-H_2s_ are capable of inducing C-H bond formation. We note that similarly prepared cTiNTs have been shown to be photochemically active for carbon dioxide reduction, albeit at much lower yields [10]. How, then, do we rationalzie this qualitative similarity in adsorption behavior but disparity in photoreduction of CO_2_? As shown by XPS results, defects on cTiNTs are substantially less than on the TiNTs. Defects such as oxygen vacancies and associated Ti^3+^ sites promote significant surface carboxylate formation due to CO_2_ adsorption, which is, in turn, postulated to be a critical intermediate in the photoconversion of CO_2_ [12].

Figure 8 shows a blow-up of the 1800–4000 cm^−1^ region which exhibits a weak “negative absorption” between ~2500 and ~3500 cm^−1^. Bicarbonate formation must involve the interaction of CO_2_ with an H-containing moiety. The negative absorption due to the depletion of moieties present in the background spectrum is consistent with the formation of bicarbonates and indicates a depletion of surface adsorbed water. The lower frequency OH stretch region is due to OH stretches of H-containing moieties within the cTiNTs. It is also possible that CO_2_ and water compete for the same surface sites and this competition leads to the depletion of OH bond-containing moieties that absorb between ~2500 and 3500 cm^−1^.

## 4. Experimental Section

### 4.1. Synthesis of TiNT, cTiNT, and in Situ Pretreatments

Titania nanotubes were prepared by a modified hydrothermal method that has been previously reported [1,2,40]. In a typical experiment, 2 g of anatase titania powder (purity 99%, Sigma Aldrich Chemicals, St. Louis, MO, USA) were stirred with 50 mL of 10 M NaOH solution (purity 97%, BDH Chemicals, Radnor, PA, USA) in a closed 125 mL Teflon cup. The Teflon cup is sealed inside a stainless steel outer vessel and placed in an oven for 48 h at 120 °C. The resulting precipitate was washed with 1 M HCl (purity 38%, EMD Chemicals, Billerica, MA, USA) followed by several washings with deionized water to attain a pH between 6 and 7. The TiNT powder thus formed was dried overnight in an oven held at 110 °C. Some of the TiNT samples were then calcined at a temperature of 650 °C to probe the effect of temperature on the morphology of these materials. The TiNTs were initially heated *in situ* under an O_2_ atmosphere at 350 °C [O_2_ ~2 Torr] to clean the surface, and were then allowed to cool to room temperature under vacuum. The TiNTs that are only heated to 350 °C are labeled by the pretreatment (*i.e.*, -TiNT-O_2_ for oxidized TiNTs) while those heated to 650 °C are labeled as cTiNT and the pretreatment (*i.e.*, -cTiNT-O_2_). After oxidative pretreatment, some of the TiNTs were treated in a H_2_ atmosphere at 350 °C [H_2_ ~2 Torr] for 3 h and then allowed to cool to room temperature under vacuum. The TiNTs heated to 350 °C that were reduced are referred to as TiNT-O_2_-H_2_ while those heated to 650 °C and then reduced after oxidation are referred to as cTiNT-O_2_-H_2_.

### 4.2. Characterization of the TiNTs

The morphology of titania nanomaterials was probed by transmission electron microscopy (TEM, STEM JEOL-2100F, Peabody, MA, USA), with an accelerating voltage of 200 kV. To establish crystallinity and phase purity, the powder X-ray diffraction (XRD) patterns of the TiNT and the cTiNT were recorded on a Rigaku Domex diffractometer (The Woodlands, TX, USA) using Cu Kα radiation, a continuous scan, and a scintillation-type detector for 2θ from 5° to 90°. X-ray photoelectron spectra (XPS) were obtained with an Omicron (Houston, TX, USA) ESCA-2000-125-based spectrometer using an Al Kα radiation source (1486.6 eV, 30 mA × 8 kV). These spectra provided data on the oxidation states of the ions in the TiNT samples. The C 1s response at 284.6 eV was used as an internal reference for the absolute binding energy.

### 4.3. In Situ FT-IR Spectroscopy

*In situ* FT-IR spectra were recorded with a Nicolet (Madison, WI, USA) 6700 FTIR spectrometer equipped with both a mercury cadmium telluride (MCT) and a DTGS (Deuterated Triglycine Sulfate) detector. Each spectrum was obtained by averaging 64 scans at a resolution of 4 cm^−1^. The custom fabricated infrared cell, which was designed to study highly scattering powder samples in a transmission mode, has been described previously [31]. Briefly, it consists of a stainless steel cube with two CaF_2_ windows positioned on opposite sides of the cube. For this study, samples were pressed onto a highly transmissive tungsten wire grid held between two nickel jaws. The grid, which is resistively heated to a temperature measured by a Chromel-Alumel thermocouple attached to its center, provides a support for highly scattering samples so that very thin samples can be studied in transmission mode. The vacuum system was pumped using a Turbo pump backed by a mechanical forepump to achieve a base pressure of 1 × 10^−5^ Torr. The infrared beam was directed out of the spectrometer, allowed to pass through the cell windows and the sample on the wire grid, and was detected with the MCT detector. The cell and detector were contained in an enclosure that was purged with boiled off nitrogen before acquisition of spectra. Unless otherwise stated, background spectra are of the samples cooled to ambient temperature, under vacuum, after pretreatment. A Baratron capacitance manometer was used to monitor the pressure of CO_2_. D_2_O, used in some of the IR experiments, was specified as 99% atomic purity.

## 5. Conclusions

This research probes the interaction of water on the cTiNTs and we propose an explanation for the unusual low frequency OH stretching region. On exposure to water the OH stretching region of c-TiNT-O_2_, illustrated in Figure 6, is broader than that reported for titania powder and what is typical for other metal oxides [41,42], extending below 3000 cm^−1^. Interaction with D_2_O demonstrates that the OH region contains exchangeable protons, and absorptions due to the deuterated analogs of the species are observed with absorptions between ~2700 and ~1950 cm^−1^. Taking into account that variations in the baseline of the spectra produce uncertainty in the extent of these absorptions, the shifts in the absorptions on deuteration are consistent with expectations based on typical frequency ratios for OH *vs.* OD stretches. Based on these observations and data in the literature, we suggest that the unusually low frequency OH stretching modes are best explained by hydrogen bonding involving H-containing moieties within the structure of the TiNTs. It is recognized that the addition of water can increase the efficiency of photoinduced reduction reactions taking place on titania [43], and proton sources are needed for the formation of hydrocarbon products. We note that the present results suggest that proton sources can be sequestered on and within the structure of the TiNTs and c-TiNTs.

Exposure of the c-TiNT-O_2_-H_2_ to CO_2_ leads to the formation of new species on the c-TiNTs, which are identified as carbonates and bicarbonates, as well as formic acid. Though there are differences in the number and nature of the carbon-containing species observed on the surface of the c-TiNT-O_2_-H_2_ *versus* the TiNT-O_2_-H_2_, many moieties are observed on both types of TiNTs. The large decrease in surface area that occurs on the collapse of the TiNTs to c-TiNTs would be expected to contribute to a smaller amount of surface carbonates and bicarbonates. However, the fact that many of the surface moieties are the same for the c-TiNT-O_2_-H_2_ and the TiNT-O_2_-H_2_ suggests that many of the sites of interaction on TiNT-O_2_-H_2_ remain on c-TiNT-O_2_-H_2_, but despite adsorption, CO_2_ photoreduction is low on the cTiNTs. We suggest that adsorption mediated by specific defects (O-vacancy and Ti^3+^), which are largely absent on cTiNT, is required for the significant photoreduction of CO_2_. An implication of these findings, then, is that a material’s defect structure is crucial for CO_2_ photoconversion.
